# Antibacterial effect of a dental adhesive containing SCH-79797 on *Streptococcus mutans* biofilms

**DOI:** 10.3389/fcimb.2026.1808368

**Published:** 2026-06-29

**Authors:** Gaozhe Zheng, Lingjun Zhang, Haiyun Dong, Yunhe Shao, Shuli Deng, Yan Sun, Yihuai Pan, Min Wang

**Affiliations:** 1 School and Hospital of Stomatology, Wenzhou Medical University, Wenzhou, China; 2 Stomatology Hospital, School of Stomatology, Zhejiang University School of Medicine, Zhejiang Provincial Clinical Research Center for Oral Diseases, Hangzhou, China

**Keywords:** biofilms, dental adhesive, dental caries, SCH-79797, *Streptococcus mutans*

## Abstract

**Objectives:**

Antibacterial modification of dental adhesives has emerged as a viable strategy for caries prevention. In this study, a newly identified dual-target antimicrobial, SCH-79797, known for its strong antibiofilm activity, was incorporated into a dental adhesive. Its antibiofilm performance was subsequently evaluated using a *Streptococcus mutans* (*S. mutans*) biofilm model.

**Methods:**

The bonding performance, polymerization behavior and surface wettability of the SCH-79797–modified adhesive were evaluated by shear bond strength testing, degree of conversion, and water contact angle measurements, respectively. Biocompatibility was examined in L929 fibroblasts using CCK-8 assays and live/dead cell staining. Antibacterial activity against biofilms was evaluated through CFU counting, MTT assays, SEM observation, and live/dead bacterial staining. In addition, biofilm virulence was evaluated by analyzing water-insoluble polysaccharide and extracellular polysaccharide (EPS) production, as well as changes in pH and lactic acid production.

**Results:**

Incorporating SCH-79797 at concentrations up to 0.5% did not adversely affect the adhesive properties and resulted in minimal cytotoxicity. Compared with the control, the SCH-79797-modified adhesive reduced bacterial viability by approximately 6 log CFU, and significantly decreased lactate production and EPS synthesis by approximately 90% and 90%, respectively.

**Conclusions:**

These findings indicate that SCH-79797-modified dental adhesives possess promising *in vitro* anticaries activity against cariogenic bacteria.

## Introduction

Dental caries is a prevalent oral disease affecting a large proportion of the global population (Bawaskar and Bawaskar, 2020). Resin-based restorative materials, including composite resins and dental adhesives, are widely used in clinical practice; however, a substantial proportion of restoration failures have been attributed to secondary caries (Tennert et al., 2024). In the United States alone, more than USD 5 billion is spent annually on the replacement of failed resin restorations (Liang et al., 2018). Clinical observations consistently indicate that secondary caries tend to originate at the resin–dentin junction, a structurally compromised zone that largely dictates the service life and clinical reliability of resin restorations (Van Meerbeek et al., 2003). The structural integrity of this junction is governed primarily by the functional performance of the adhesive. In this context, a long-term prospective study revealed that nearly 50% of Class II composite restorations failed because secondary caries developed along the margins of the adhesive interface (van Dijken and Pallesen, 2014). Accumulating evidence indicates that cariogenic microbial communities, particularly biofilms enriched with *Streptococcus mutans (S. mutans)*, are involved in the biological processes underlying secondary caries (Mokeem et al., 2022; Qiu et al., 2025). These biofilms compromise the bonding interface by producing lactic acid, extracellular polysaccharides (EPS), and other metabolites in combination with water and salivary enzymes, leading to the degradation of adhesive monomers (Cocco et al., 2015; Leyva Del Rio et al., 2020). Moreover, in deep cavities, residual bacteria are often intentionally preserved to avoid pulp exposure, which may further jeopardize the longevity of resin restorations (Yu et al., 2017). Thus, antibacterial modification of dental adhesives has gained attention as a viable method for maintaining interfacial integrity and suppressing secondary caries (Li et al., 2022).

Antimicrobial dental adhesives have been developed to reduce cariogenic bacterial accumulation at the tooth–restoration interface, and previous studies have demonstrated that such modifications effectively decrease bacterial colonization along restoration margins (Figueiredo Macedo de Lima et al., 2020). From a compositional perspective, the antibacterial approaches currently applied in dental adhesives can be divided into three main categories: synthetic antibacterial agents, naturally derived compounds, and inorganic additives (Sousa et al., 2024). With respect to synthetic antibacterial systems, compounds such as chlorhexidine and quaternary ammonium compounds have been integrated into commercially available dental adhesives. Adhesives containing quaternary ammonium moieties generally demonstrate more persistent antimicrobial effects, whereas chlorhexidine-based formulations often exhibit diminished long-term efficacy because of drug leaching and potential interference with resin polymerization (Altintop et al., 2025); (Leyva Del Rio et al., 2020); (Muratovska et al., 2018); (Bijle et al., 2020). Nevertheless, recent studies have suggested that repeated or long-term use of chlorhexidine and quaternary ammonium compounds may contribute to reduced bacterial susceptibility, highlighting a potential risk of antimicrobial resistance (Wang et al., 2017). Natural compounds, such as chitosan, epigallocatechin gallate, and other plant-derived agents, are attractive antibacterial additives because of their favorable biocompatibility and antimicrobial potential; however, their efficacy is highly concentration-dependent, and excessive incorporation may compromise adhesive performance (Hass et al., 2025). Inorganic additives, particularly metal nanoparticles, can impart broad-spectrum antibacterial activity; such approaches are still associated with safety concerns and may adversely influence the structural integrity and durability of dental adhesive systems (Wang et al., 2023). Taken together, despite substantial progress, achieving an optimal balance among antibacterial efficacy, bonding durability, and biocompatibility remains a major challenge for current antimicrobial adhesive strategies (Sousa et al., 2024). Therefore, further modification strategies and the exploration of alternative antibacterial agents are warranted.

Consistent with this need, SCH-79797 has recently attracted attention as a novel antimicrobial molecule featuring a dual-target mode of action that targets both bacterial folate pathways and compromises membrane integrity. The compound SCH-79797, chemically classified as a pyrroloquinazoline analogue (N3-cyclopropyl-7-{[4-(1-methylethyl)phenyl]methyl}-7H-pyrrolo[3, 2-f]quinazoline-1, 3-diamine), was initially developed in 1999 (Ahn et al., 1999). It has been widely utilized as a selective inhibitor targeting protease-activated receptor-1 (PAR-1) (Zhang et al., 2017). A recent published study demonstrated that SCH-79797 possesses robust broad-spectrum antibacterial activity against diverse bacterial species spanning both gram-positive and gram-negative groups, such as *Enterococcus faecalis*, *Neisseria gonorrhoeae*, and *Staphylococcus aureus* (Martin et al., 2020). Notably, this mechanism confers superior therapeutic efficacy compared with conventional combination antibiotic therapies and remains effective against persistent and drug-resistant bacterial populations (Martin et al., 2020). In our previous studies, SCH-79797 markedly inhibited *S. mutans* biofilm formation, accompanied by significant reductions in lactic acid production and EPS synthesis (Zhang et al., 2022b). These outcomes were attributed to the suppression of bacterial growth and the regulation of genes related to polysaccharide synthesis, acid production, biofilm formation, quorum sensing, and interspecies competition (Zhang et al., 2022b). Moreover, SCH-79797 selectively inhibited cariogenic *S. mutans* while preserving the commensal *Streptococcus sanguinis* within dual-species biofilms (Zhang et al., 2025). In addition, pronounced reductions were observed in dual-species biofilm accumulation, microbial metabolic function, extracellular matrix polysaccharide production, and acidogenic potential, accompanied by a decreased proportion of *S. mutans* (Zhang et al., 2025). Together, these observations highlight that SCH-79797 holds substantial promise for use in caries prevention strategies.

To date, no studies have investigated the antibacterial properties of adhesive containing SCH-79797. Therefore, *S. mutans* biofilms were selected as an *in vitro* system to assess the antibacterial effect of a dental adhesive formulated with SCH-79797, with the aim of evaluating its potential to prevent secondary caries. Our hypotheses were as follows: (1) the presence of SCH-79797 in the adhesive system was anticipated to maintain adequate bonding properties without inducing notable cytotoxicity, and (2) the SCH-79797–modified adhesive would markedly inhibit *S. mutans* biofilm formation and its cariogenic virulence, thereby demonstrating a promising ability to mitigate the development of secondary caries.

## Materials and methods

### Adhesive formulation and specimen models

SCH-79797–modified Single Bond 2 adhesive formulations were prepared by incorporating SCH-79797 into Single Bond 2 adhesive (3M Company, St. Paul, MN, USA). By adjusting the proportion of SCH-79797 through sequential dilution, adhesives containing increasing concentrations of the antimicrobial agent (0–2%, w/v) were obtained following a previously established method (Freitas et al., 2021). SCH-79797 powder was initially dissolved in dimethyl sulfoxide (DMSO) to obtain a concentrated stock solution (Zhang et al., 2025). The stock solution was then incorporated into the adhesive at predefined mass fractions (e.g., 0-2%, w/v) under light-protected conditions to minimize potential photo-degradation. The mixture was vigorously vortexed for uniform dispersion and subsequently centrifuged to eliminate entrapped air bubbles. All procedures were performed at room temperature, and the modified adhesive was freshly prepared prior to use. These formulations were employed to assess the degree of conversion, water contact angle, and cellular biocompatibility, and the bonding performance and antibacterial properties associated with the experiments were evaluated using Molds 1 and 2. The degree of conversion was assessed by applying a small volume of uncured adhesive onto the ATR crystal to collect the precuring spectrum, followed by air thinning, film coverage, and photoactivation. For contact angle analysis, the adhesives were coated onto glass slides and the adhesive-coated glass slides were light-cured for 20 s using a LED curing unit (1200 mW/cm^2^; Bluephase, Ivoclar Vivadent, Schaan, Liechtenstein) (Yildirim-Manav et al., 2025). To determine the bonding performance and antibacterial properties of SCH-79797-modified dental adhesives, two standardized specimen models (Molds 1 and 2) were established.

#### Mold 1

Dentin–resin bonding model for shear bond strength evaluation. Sound human maxillary third molars of humans were provided by the Affiliated Stomatology Hospital of Wenzhou Medical University (Ethical approval: WYKQ2021013). Enamel was removed from both the mesial and distal sides to generate standardized flat dentin areas, which were standardized by sequential polishing with 200-, 400-, and 600-grit silicon carbide papers (M&G Stationery Inc., Shanghai, China). Following adhesive treatment, resin composite cylinders were formed on the dentin surfaces with the aid of a rubber mold (4 mm in diameter × 4 mm in height; M&G Stationery Inc., Shanghai, China). This model setup served to evaluate the dentin shear bond strength (Su et al., 2018). In brief, the dentin surfaces underwent acid etching and were treated with the adhesive, air-thinned, and light-cured prior to the fabrication of the resin composite cylinders.

#### Mold 2

Resin disk model for antibacterial evaluation. Resin disk substrates were prepared using an acrylic plate molding system. Briefly, a 2-mm-thick acrylic plate (M&G Stationery Inc., Shanghai, China) was perforated with an 8-mm diameter punch to create disk-shaped cavities. The resin was packed into the cavities between acrylic plates to obtain standardized resin disks. The surface was carefully flattened using a glass slide to obtain a uniform surface. Specimens were then light-cured using a dental curing unit (light intensity: 1200 mW/cm^2^), for 20 s. After curing, the specimens were removed from the mold and stored under specified conditions prior to further experiments (Desai and S M, 2024). After curing, the disks were coated with the respective SCH-79797–modified adhesives and used as substrates for *in vitro* antibacterial assays (Esteban Florez et al., 2018).

### Dentin shear bond strength test

Dentin bonding performance was evaluated by shear bond strength testing (SBS) to assess the effects of different concentrations of SCH-79797–modified adhesives on dentin bonding performance using the dentin–resin bonding model (Mold 1). SBS was measured as previously described (Zhang et al., 2015b), with slight modifications. Shear testing was conducted on samples following 24 h of water storage at 37 °C, using a universal mechanical tester (Instron, Norwood, MA, USA). A chisel-shaped loading head was positioned as close as possible to the adhesive interface, and load was applied at a crosshead speed of 0.5 mm/min until failure occurred. The shear bond strength values were calculated by dividing the maximum load at failure (N) by the bonded surface area (mm²) and expressed in megapascals (MPa). Six specimens were tested per group.

### Water contact angle and degree of conversion analysis

Assessment of the physicochemical behavior of SCH-79797–modified adhesives typically involves analysis of surface wettability and monomer conversion (Bottino et al., 2013; Esteban Florez et al., 2018). The wettability and curing efficiency of the adhesive surfaces were examined through contact angle measurements and FTIR analysis (FTIR, Agilent Technologies, Santa Clara, CA, USA).

For contact angle analysis, following the placement of a 5 μL deionized water droplet on the cured surface, contact angle images were acquired after a 10-s equilibration period. (Attension Theta, Biolin Scientific, Gothenburg, Sweden). The static contact angle was analyzed, and five independent readings were obtained per sample.

Attenuated total reflectance (ATR) with FTIR spectroscopy was employed to quantify the monomerconversion of the adhesive. After gentle air-thinning for 30 s, the adhesive was sealed with a polyester film and photopolymerized for 20 s, after which the postcuring spectrum was acquirce methacrylate C=C bonds (1637 cm^-1^) and ester C=O bonds (1715 cm^-1^) were analyzed. The calculation of DC was performed on the basis of the following formula:


$$\text{DC }\left(\%\right)\;=\;[1-\left(1637/1715\text{ after curing}\right)/\left(1637/1715\text{ before curing}\right)]\;\times \;100\%$$

### SCH-79797 release

Mold 2 specimens were immersed in 2 mL of distilled water at 37 °C for 1, 3, and 5 days, respectively. The release of SCH-79797 was quantified using a UV-vis recording spectrophotometer (U-2010 Hitachi, Tokyo, Japan) by measuring the peak height from an arbitrary baseline at 341 nm, which corresponds to the characteristic absorption of SCH-79797. Three samples were tested for each condition, and the reproducibility was ensured by performing triplicate UV spectrometry measurements for each sample.

### Cytotoxicity evaluation

Cytotoxicity evaluation is essential for assessing the biocompatibility of SCH-79797–modified adhesives. Mouse fibroblast L929 cells were used to evaluate the cytotoxicity of the cured adhesives specimens using Cell Counting Kit-8 (CCK-8) assays and live/dead fluorescence staining (Zhang et al., 2021; Zhao et al., 2021).

For the CCK-8 assay, Mold 2 specimens were immersed in 2 mL of DMEM supplemented with 10% fetal bovine serum and 1% penicillin–streptomycin and incubated at 37 °C for 24 h to obtain the extract media. Briefly, L929 cells were seeded into 96-well plates at a density of 8 × 10³ cells/well and cultured for 24 h. Subsequently, 10 μL of CCK-8 solution was added to each well containing 100 μL of culture medium. The cells were then incubated at 37 °C for 1 hour in the dark, and the absorbance was measured at 450 nm using a microplate reader. Cell viability was calculated relative to that of the untreated control group.

For live/dead staining, L929 cells were seeded into 24-well plates at a density of 5×10^4^ cells per well and cultured in complete medium containing serum at 37 °C in a 5% CO_2_ incubator for 24 hours to allow cell attachment. After attachment, the original medium was aspirated and replaced with the corresponding extract medium for an additional 24 hours. Following the treatment, the extract medium was aspirated, and the cells were gently washed with PBS for 5 minutes. The live/dead staining working solution was prepared according to the manufacturer’s instructions: 10 μL of Calcein-AM (1000×) and 10 μL of PI (1000×) were added to 10 mL of assay buffer (or PBS) to obtain 10 mL of Calcein-AM/PI working solution. Then, 150 μL of the working solution was added to each well and incubated at 37 °C for 30 minutes in the dark. After incubation, the working solution was aspirated, and the cells were gently washed three times with PBS (being careful not to detach the cells). Finally, the cells were observed under a fluorescence microscope (Carl Zeiss Microscopy GmbH, Jena, Germany), where live cells displayed green fluorescence and dead cells displayed red fluorescence.

### Bacterial strains, growth conditions and biofilm formation

In the experiments, *S. mutans* UA159 (provided by the Institute of Stomatology, School and Hospital of Stomatology, Wenzhou Medical University) was primarily utilized. For bacterial proliferation, Brain Heart Infusion (BHI) broth (Oxoid, Basingstoke, UK) was used. For the biofilm-related studies, a sucrose-enriched brain heart infusion medium (BHIS, containing 1% sucrose) was used. The cells were incubated at 37 °C in an environment supplemented with 5% CO_2_. Adhesive samples (Mold 2) used for colony forming units (CFUs), MTT assay, scanning electron microscopy (SEM) imaging, live/dead bacterial staining, anthrone assay, bacterial/EPS staining, measurement of pH and Lactic acid production were placed in 24-well plates (three specimens per group) with the adhesive surface facing upward. Each well was filled with 2 mL of BHIS medium containing *S. mutans* at a final concentration of 1 × 10^6^ CFU/mL. Biofilm development was promoted by culturing the plates for 24 h at 37 °C in an atmosphere supplemented with CO_2_ (Xu et al., 2025).

### CFUs

Colony-forming unit analysis was conducted to evaluate bacterial proliferation and determine the viability of bacteria within the biofilms by counting the colonies that developed the SCH-79797–modified adhesive specimens. The samples (Mold 2) were PBS-rinsed to remove planktonic bacteria after biofilm formation. Biofilms were mechanically scraped from the surface of the samples, resuspended in PBS, and vortexed. The suspension was then centrifuged, the supernatant was discarded, and the bacterial pellet was resuspended in PBS followed by vortexing to obtain a homogeneous bacterial suspension before serial dilution. The suspensions were spread onto BHI agar plates, followed by incubation for 48 h, after which viable colonies were enumerated and expressed as CFU per sample (Chen et al., 2020).

### MTT assay

The metabolic viability of the biofilms was quantified through MTT-based analysis (Kraigsley et al., 2012). After 24 hours of biofilm formation, the samples (Mold 2) were gently washed with PBS and transferred to fresh 24-well plates. Following the addition of MTT solution (0.5 mg/mL, 1 mL), the mixture was incubated at 37 °C for 1 h. The MTT reagent was removed and replaced with 1 mL of DMSO, followed by a further 20-min incubation period. The absorbance at 540 nm was measured after 200 μL of each sample was added to 96-well plates.

### SEM imaging

The ultrastructural features of bacteria in surface-associated biofilms were characterized using SEM (Yu et al., 2017). The samples (Mold 2) with adhered biofilms were fixed in 2.5% glutaraldehyde at 4 °C overnight. After fixation, samples were dehydrated through sequential ethanol gradients (30%, 50%, 70%, 90%, 100%), air-dried for 6 hours, and sputter-coated with gold prior to observation. Samples were sputter-coated with gold using an ion sputter at 15 mA for 80 seconds, resulting in a coating thickness of approximately 10–15 nm. SEM images were acquired using a SU8010 (SU8010, Hitachi, Tokyo, Japan) at an accelerating voltage of 5 kV, working distance of 8–10 mm, spot size of 30, and using a secondary electron detector (SE2). Magnifications of 1000× and 5000× were used for imaging.

### Live/dead bacterial staining

The purpose of live/dead staining was to evaluate the effectiveness of SCH-79797–modified adhesive specimens (Mold 2) on biofilms (Zhang et al., 2022b). To distinguish live and dead bacteria, biofilms were treated with SYTO 9 and propidium iodide (each at 2.5 μM) for 30 min and subsequently examined under a confocal laser scanning microscope (Nikon A1, Nikon Corporation, Japan) using a 60× objective. Viable bacteria appeared green, whereas nonviable bacteria were observed in red.

### Anthrone assay

Quantification of water-insoluble glucans (WIG) was performed using the anthrone method. Studies of WIGs can help determine how well certain SCH-79797-modified adhesive samples (Mold 2) can inhibit or break down the biofilm matrix, reducing bacterial adhesion and preventing caries (Zhang et al., 2022a). After the biofilms were washed with PBS and resuspended in 0.4 M NaOH, they were centrifuged, after which a 100-μL aliquot of the supernatant was reacted with 300 μL of anthrone reagent at 95 °C for 6 min. The WIG content was calculated from the absorbance values measured at 625 nm with reference to a standard curve. A standard curve was generated using D-glucose (0, 10, 25, 50, 100, 200 µg/mL) dissolved in 0.4 M NaOH. The anthrone reaction was performed as described for samples: 100 µL of each standard or sample was reacted with 300 µL of anthrone reagent at 95 °C for 6 min. Absorbance was measured at 625 nm, and the glucose concentration was plotted against absorbance. Water-insoluble glucan (WIG) content was expressed as glucose equivalents (µg) per sample.”.

### Bacterial/EPS staining

Bacteria/EPS dual staining is used to study biofilms, particularly to differentiate between bacterial cells and the EPS that they secrete (Klein et al., 2009). For bacteria/EPS dual staining, adhesive specimens (Mold 2) were incubated with *S. mutans* (1 × 10^6^ CFU/mL) in BHIS medium supplemented with 2.5 μM Alexa Fluor 647-dextran (Molecular Probes, Invitrogen Corp., Carlsbad, CA, USA) for 24 hours. After incubation, the biofilms were fluorescently labeled with 2.5 μM SYTO 9 and observed using confocal laser scanning microscopy (CLSM).

### Measurement of pH and lactic acid production

Lactic acid production and pH experiments were conducted to reveal the acidic capacity of biofilms under the influence of SCH-79797-modified adhesive samples (Mold 2) (Cheng et al., 2012). Supernatant pH values were assessed with a digital pH measurement device. (Shanghai Leici Instrument Co., Ltd., Shanghai, China). The blank group represented the pH of the fresh medium, while the experimental groups represented the pH after 24 hours of biofilm incubation.

For lactic acid production, biofilms were washed with CPW solution (prepared by adding L-cysteine hydrochloride, sodium chloride, peptone, yeast extract, and 80% glycerol to deionized water, after which the pH was adjusted to 7.3 and sterilizing at 121 °C) and incubated with BPW solution (prepared by dissolving BPW powder and sucrose in deionized water, adjusting the pH to 7.2, and sterilizing at 121 °C) for 3 hours at 37 °C. The supernatants were mixed with the reaction solution (1 M glycine and 0.8 M hydrazine sulfate, pH 9.5 were first mixed to obtain mixed solution. Subsequently, 26 mM NAD^+^ was added to mixed solution at a ratio of 20 mL to 2.35 mL to prepare the reaction solution), and the absorbance was measured at 340 nm before (A1) and after (A2) the addition of lactate dehydrogenase. Lactic acid production was calculated as ΔA (A2 − A1) on the basis of a standard curve.

### Statistical analysis

All experiments were performed independently at least three times. Data are presented as the mean ± standard deviation (SD). Prior to statistical analysis, the normality of the data distribution and the homogeneity of variance were evaluated. One-way analysis of variance (ANOVA) was used to determine statistical differences among groups. A value of *P*< 0.05 was considered statistically significant. Statistical differences were evaluated using letter-based annotations; groups with identical letters showed no significant differences (*P* ≥ 0.05), whereas groups with different letters differed significantly (*P*< 0.05).

## Results

### The preparation of SCH-79797-modified adhesives and the two specimen models (Mold 1 and Mold 2)

**Figure 1:**
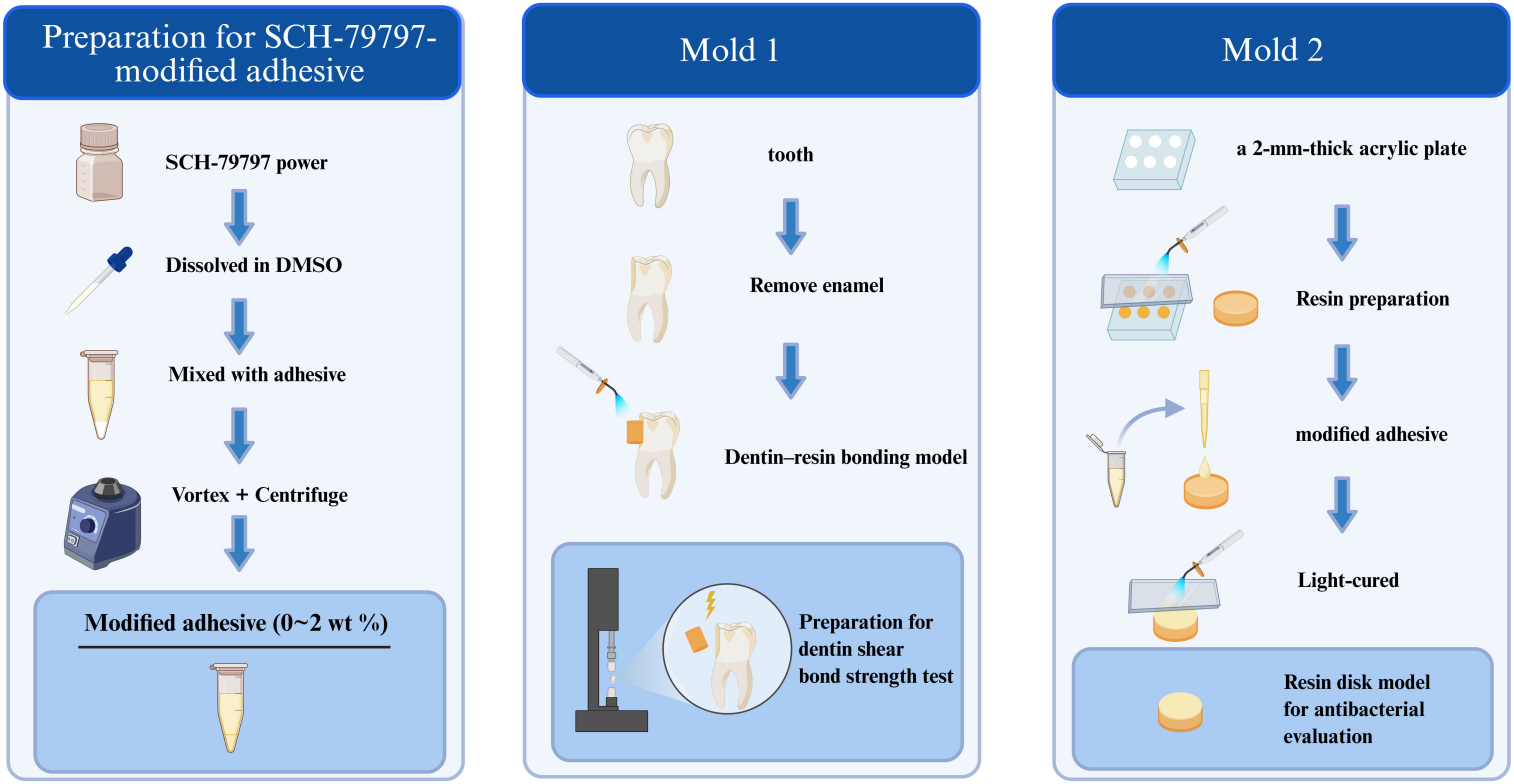
Schematic diagram of the preparation process for SCH-79797-modified adhesive and the two specimen models (Mold 1 and Mold 2). Citation: Created in BioRender. Zheng, G. Z. (2026) https://BioRender.com/xku5gvc License: BioRender Academic Publication License - Open Access sublicensing under CC-BY 4.0 and more restrictive models, subject to compliance with BioRender's Terms of Service and Academic License Terms (including no commercial use without an Industry Plan). Source: BioRender (Science Suite Inc. dba BioRender) - Confirmation of Publication and Licensing Rights issued March 23, 2026 (Agreement No. UP29IC28GU). Material status: Adapted (modifications were made to the original Completed Graphic).

To facilitate understanding of the experimental workflow, a schematic illustration summarizing the preparation of SCH-79797-modified adhesives and the two specimen models (Mold 1 and Mold 2) has been provided (**Figure 1**).

### SCH-79797 incorporation does not affect the bonding performance, degree of conversion, or surface wettability of dental adhesives

**Figure 2:**
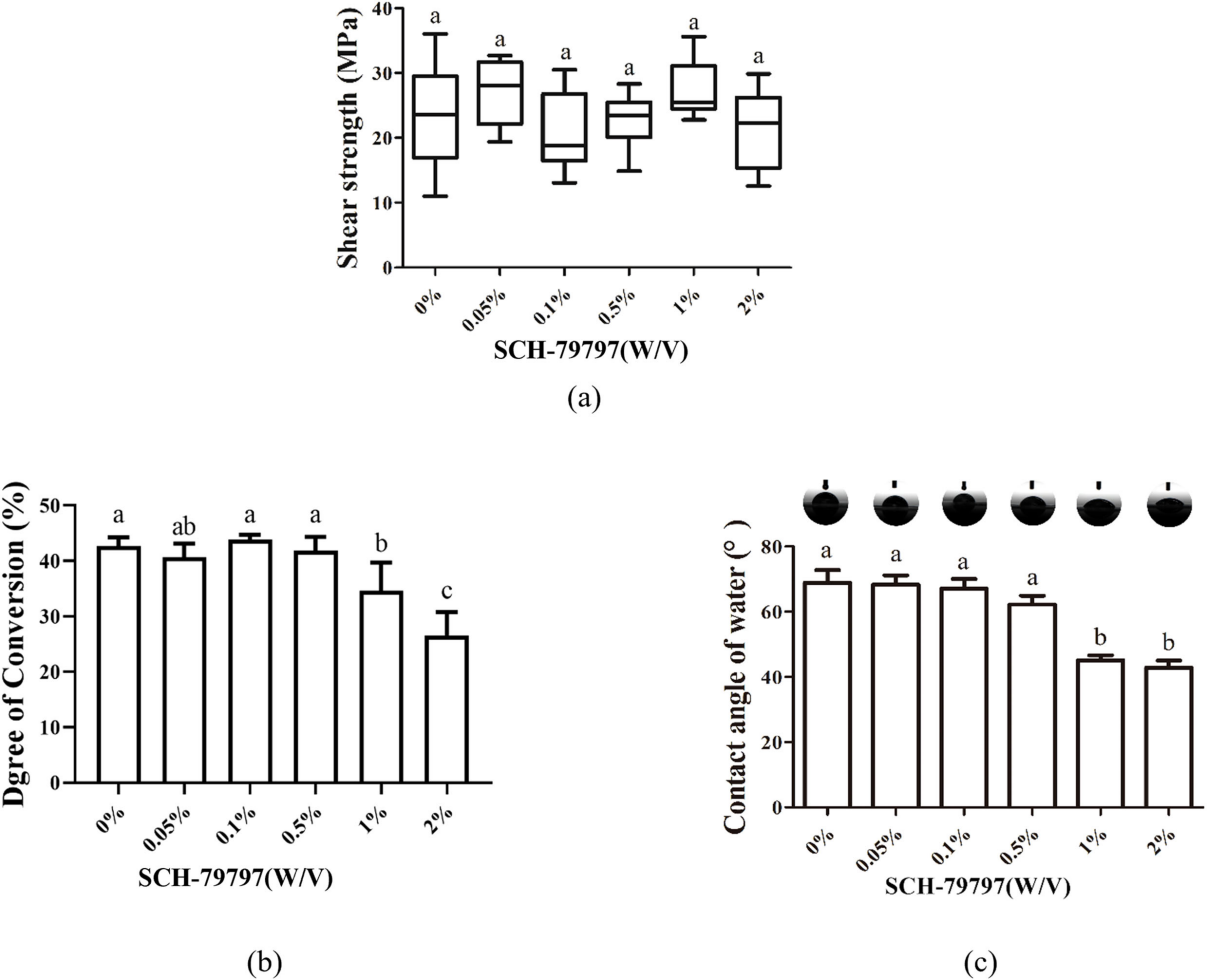
Bonding performance and polymerization-related properties of SCH-79797–modified adhesives at different concentrations. **(a)** Dentin shear bond strength (SBS) of adhesives containing different concentrations of SCH-79797 after 24 h of water storage; **(b)** monomer double bond conversion rate of SCH-79797-modified adhesive; **(c)** water contact angle of the cured adhesive surfaces. Data are presented as mean ± SD. The results are expressed as the means with corresponding standard deviations, and distinct alphabetical symbols represent significant differences between experimental groups (*P*< 0.05).

The bonding performance of the SCH-79797–modified dental adhesive was first evaluated by shear bond strength testing. As illustrated in **Figure 2a**, the shear bond strength values of the modified adhesive were comparable to those of the control group, suggesting that the presence of SCH-79797 did not compromise the adhesive bonding performance (*P* > 0.05). The polymerization behavior of the modified adhesives was further assessed by measuring the degree of double-bond conversion, as presented in **Figure 2b**. Compared with the commercial control, the incorporation of SCH-79797 at low concentrations (0.05–0.5%) did not affect monomer conversion (*P* > 0.05). However, higher loadings (≥1%) resulted in a significant decrease in double-bond conversion (*P<* 0.05). Consistent with these findings, as presented in **Figure 2c**, the water contact angle displayed a comparable trend across different concentrations. Specifically, the water contact angle values for the 0.05%, 0.1%, and 0.5% SCH-79797–modified adhesives were comparable to those of the control (*P* > 0.05), whereas the water contact ang les of the 1% and 2% groups significantly decreased (*P*< 0.05).

### SCH-79797 release from molds is concentration- and time-dependent

**Figure 3:**
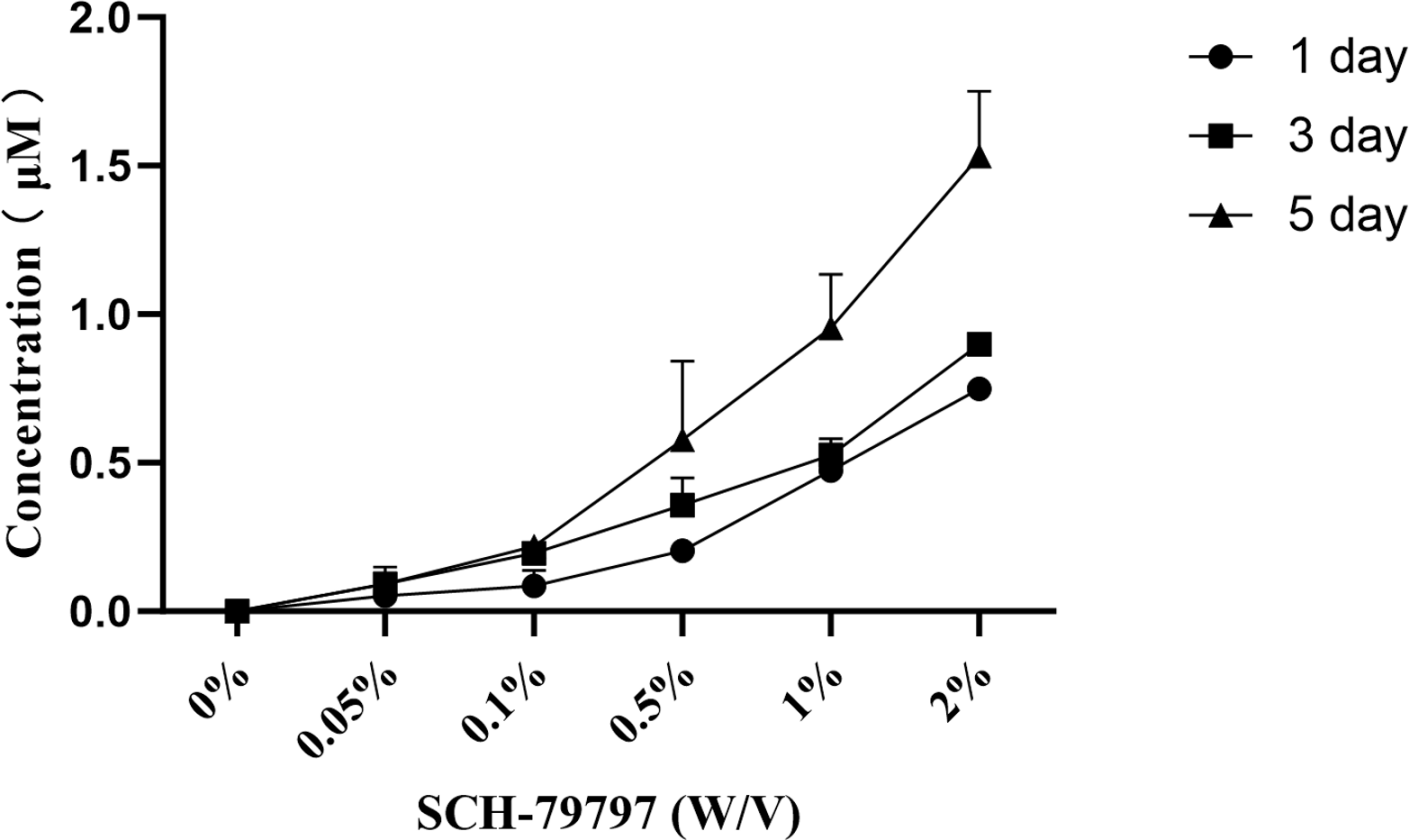
Cumulative release of SCH-79797 (μM) from molds immersed in 2 mL of distilled water at 37 °C for 1, 3, and 5 days at different initial concentrations (0%, 0.05%, 0.1%, 0.5%, 1%, 2%). Release was quantified using a UV-vis spectrophotometer at 341 nm.

As shown in **Figure 3**, the cumulative release of SCH-79797 in distilled water increased with both the initial SCH-79797 concentration and immersion time, demonstrating clear concentration-dependent and time-dependent release characteristics. Specifically, at 1 day of immersion, the release amount in all concentration groups was relatively low. When the immersion time was extended to 3 days and 5 days, the release gradually increased. Among all groups, the 2% SCH-79797 group exhibited the highest cumulative release at day 5, reaching nearly 1.50 μM. In contrast, the release amounts in the 0.05% and 0.1% concentration groups remained at low levels at all time points. Overall, the release of SCH-79797 demonstrated concentration-dependent and time-dependent characteristics.

### SCH-79797–modified adhesives exhibit low cytotoxicity

**Figure 4:**
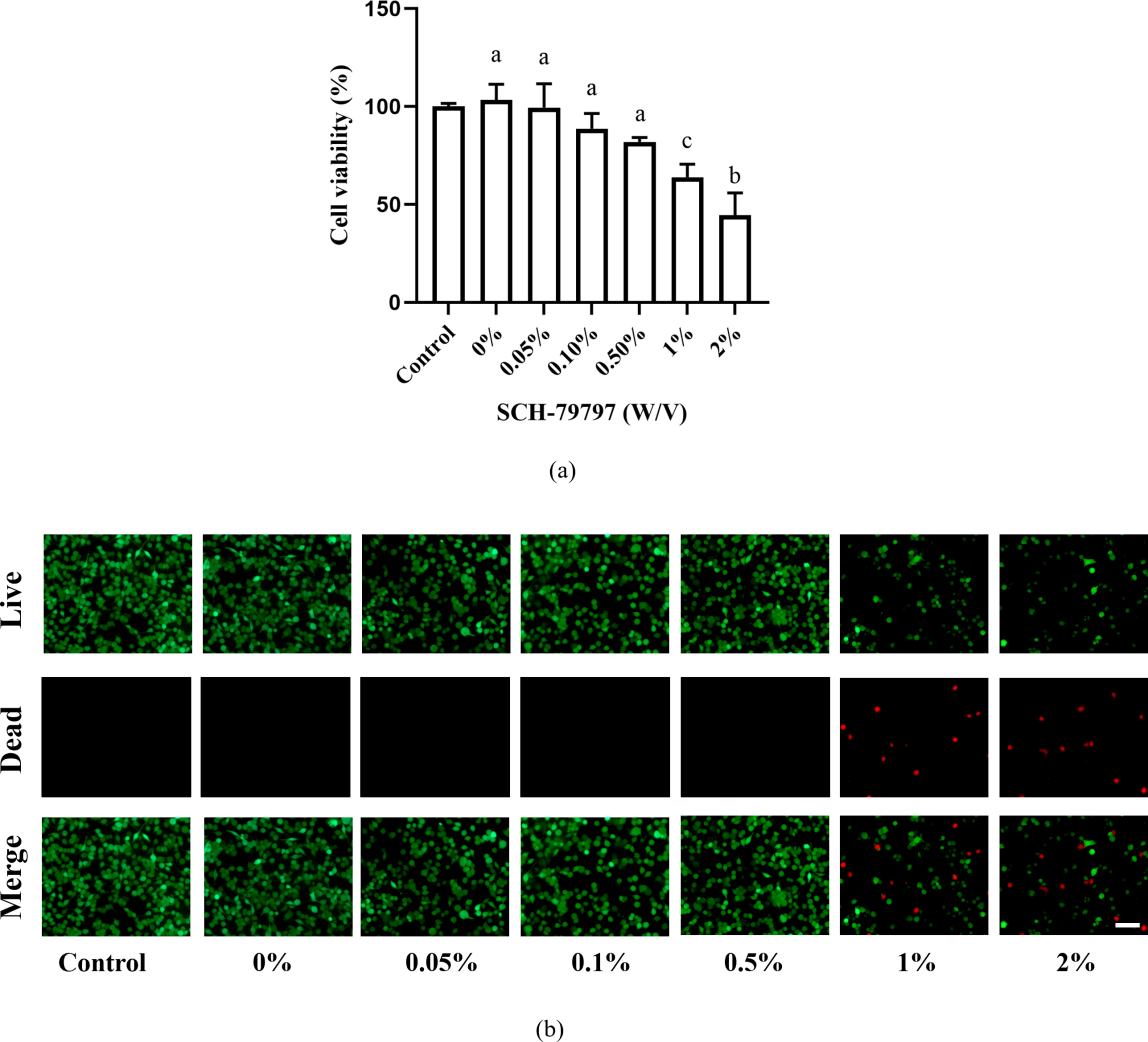
Cytotoxicity of different concentrations of SCH-79797-modified adhesive. **(a)** Survival of L929 cells after 24 h of incubation with sample extracts; the control group represents the group of cells cultured in normal medium. **(b)** Fluorescence micrographs illustrating live/dead staining of L929 cells after 24 h of sample extract treatment are presented, with live cells shown in green and dead cells in red. The objective magnification was 40×, and the scale bar is 200 μm. Data are expressed as mean ± SD (*P*< 0.05).

The cytocompatibility of SCH-79797–modified adhesives was evaluated using the CCK-8 assay. As shown in the **Figure 4a**, no significant differences in cell viability were observed between the control group and the 0-0.5% SCH-79797 groups (**Figure 4a**, p > 0.05), indicating favorable cytocompatibility at lower SCH-79797 concentrations (0%, 0.05%, 0.1% and 0.5%). In contrast, significantly reduced cell viability was observed in the 1% and 2% groups, suggesting dose-dependent cytotoxic effects at higher concentrations. These results demonstrate that SCH-79797–modified adhesives maintain acceptable biocompatibility at concentrations up to 0.5%.

According to the live/dead fluorescence staining results **Figure 4b**, when the extract concentration was below 0.5%, cells grew well and only a few red fluorescent dead cells were observed, showing no significant difference from the control group. However, when the concentration reached 1% and 2%, the number of red fluorescent dead cells increased markedly, indicating increased cell death in a concentration-dependent manner (0.5%: 0.1%, 1%: 0.5%, 2%: 1%). These findings further suggest that SCH-79797 concentrations below 0.5% exert minimal cytotoxic effects on L929 cells, whereas higher concentrations may compromise cellular viability.

### Reduced bacterial adhesion of *S. mutans* biofilms on SCH-79797–modified adhesives

**Figure 5:**
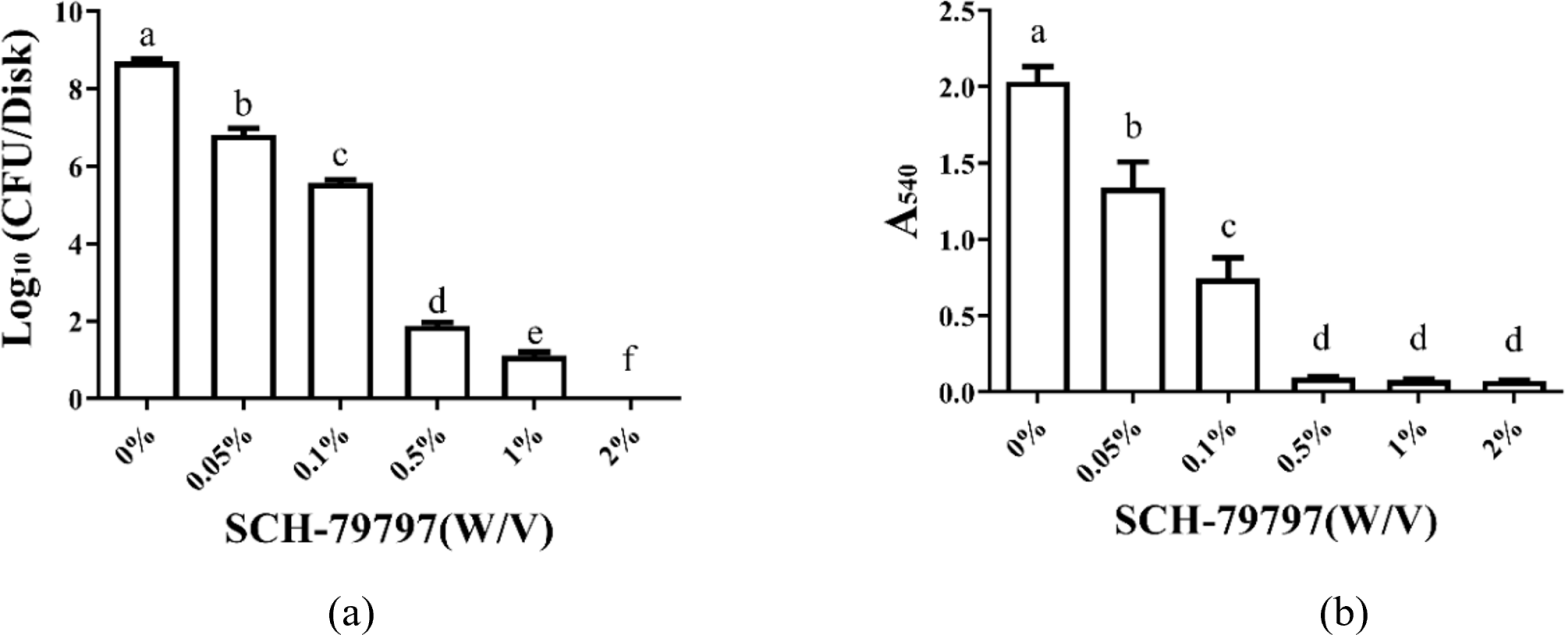
Evaluation of the *S. mutans* biofilm burden and metabolic status on adhesive samples. **(a)** CFU count of the 24 h *S. mutans* biofilm formed on adhesive samples. **(b)** MTT assay of the 24 h *S. mutans* biofilms. Data are presented as mean ± SD. The data are displayed as the means ± standard deviations; groups marked with different letters differed significantly (*P<* 0.05).

After SCH-79797 was added to the dental adhesive, the number of adhering biofilm colonies in the modified adhesive sample was much lower than that in the 0% group, and when the concentration of SCH-79797 in the adhesive reached 0.1%, the bacteriostatic rate against *S. mutans* biofilms was 99.92%. (**Figure 5a**; *P<* 0.05). In the 2% group, no bacterial growth was observed on BHI agar plates, suggesting a potent antibacterial effect. Nevertheless, because the CFU method detects only culturable bacteria, the possible presence of a small number of viable but non-culturable cells cannot be completely excluded (**Figure 5a**). Compared with that of the 0% group, the metabolic activity of the modified adhesive sample adhering to the biofilm was dramatically reduced. No discernible bacterial was detected at treatment concentrations of 0.5%, 1%, or 2% (**Figure 5b**).

### Structural alterations of *S. mutans* biofilms on SCH-79797–modified adhesives

**Figure 6:**
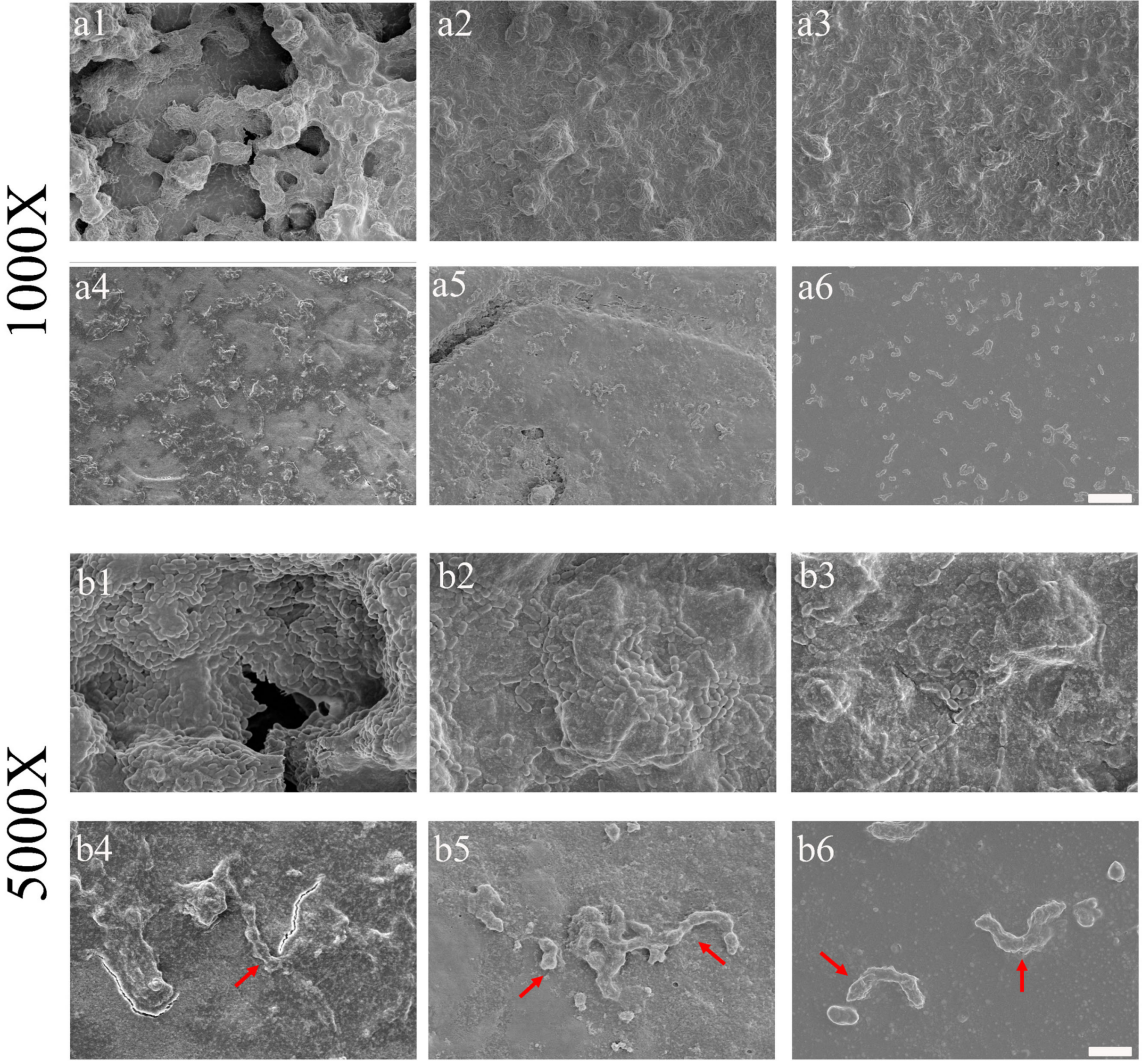
SEM examination of the *S. mutans* biofilm on each set of adhesive samples. (1–6) represent the corresponding groupings of 0%, 0.05%, 0.1%, 0.5%, 1%, and 2%, respectively. The biofilm magnified 1000 times is represented by **(a)**, with a scale bar of 50 μm; the biofilm magnified 5000 times is represented by **(b)**, with a scale bar of 10 μm.

As shown in **Figure 6**, the *S. mutans* biofilm expanded in the 0% group, with an evident three-dimensional structure and a substantial number of matrix-encapsulated bacteria. The thickness of the bacterial biofilm on the SCH-79797-modified adhesive samples (0.05%, 0.1%) decreased, and the three-dimensional structure vanished. A considerable number of matrix-wrapped bacteria could still be found. The bacteria were plump and arranged in a chain or scaffold pattern (**Figures 6a2, b2, a3, b3**). The biofilm matrix was significantly reduced in the drug-modified adhesive samples at high concentrations (0.5%, 1%, and 2%) (**Figures 6a4–a6**). Only a few bacteria were distributed singly or in chains, with reduced bacterial cell size and partial lysis, as indicated by the red arrows (**Figures 6b4–b6**).

### Reduced viability of *S. mutans* biofilms on SCH-79797–modified adhesives

**Figure 7:**
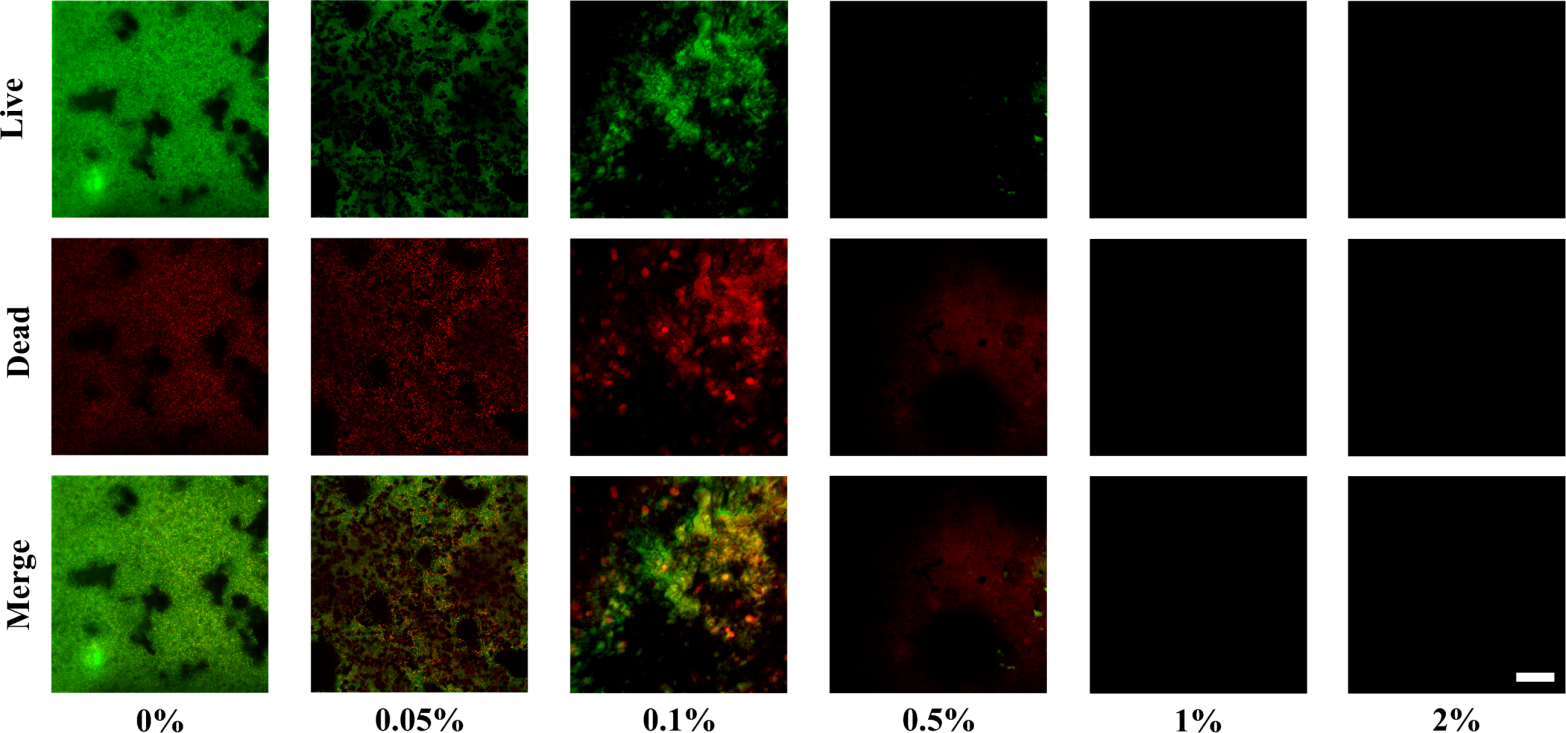
Live/dead fluorescence imaging of *S. mutans* biofilm bacteria on adhesive samples using CLSM. The objective magnification was 60×, and the scale bar is 20 μm. In contrast to dead bacteria, which display red fluorescence, viable bacteria emit green fluorescence.

The live/dead fluorescence images of *S. mutans* were shown in **Figure 7**. As the concentration of SCH-79797 in the adhesive increased, the overall bacterial load decreased dramatically. In the 0.5% group, primarily dead bacteria (red) clung to the modified adhesive sample. When the sample concentration was 1% or 2%, no apparent bacterial fluorescence was detected.

### Reduced EPS production by *S. mutans* on SCH-79797–modified adhesives

**Figure 8:**
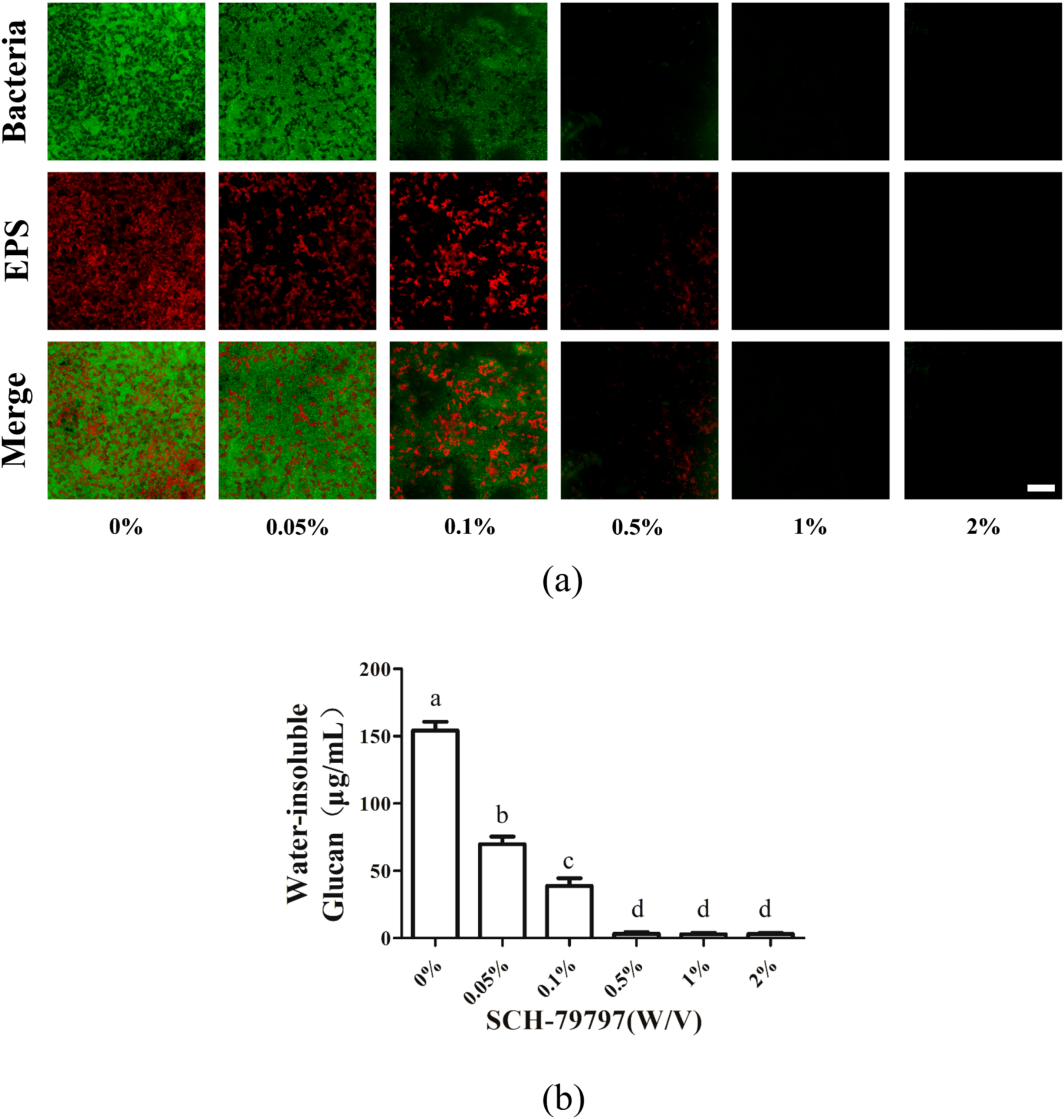
Biofilm EPS and WIG yields on adhesive samples. **(a)** Dual staining of *S. mutans* biofilms for bacteria and EPS was performed, with bacterial cells visualized in green and EPS in red. Images were acquired using a 60× objective, with a scale bar of 20 μm. **(b)** WIG yields of bacterial biofilms from each group. Values are reported as the means ± standard deviations; groups marked with different letters differed significantly (*P*< 0.05).

The amount of biofilm EPS adhering to the surface of no or low-concentration SCH-79797-modified adhesive samples (0%, 0.05%, and 0.1%) varied with the amount of bacteria, as shown in **Figure 8a**. Only small amounts of bacteria and EPS were visible at drug concentrations up to 0.5%, and when the drug concentration was further increased, no obvious bacteria or EPS fluorescence were observed. The WIG yield trend was similar to that of bacteria/EPS staining, and the WIG yield decreased in a dose-dependent manner when the SCH-79797 concentration ranged from 0%-0.1% (**Figure 8b**, *P*< 0.05). No measurable WIG was observed at drug concentrations of 0.5% or higher (**Figure 8b**, *P*< 0.05).

### Reduced acid production by *S. mutans* on SCH-79797–modified adhesives

**Figure 9:**
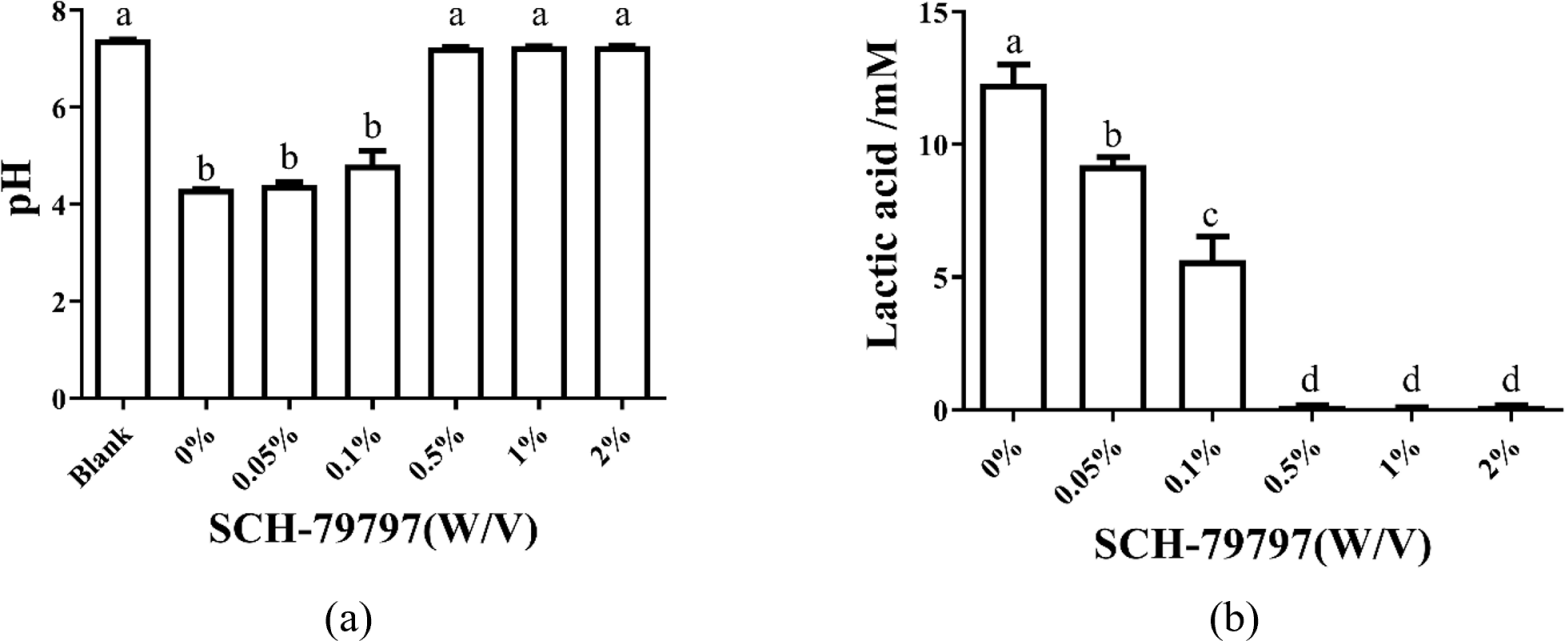
Acid production capacity of *S. mutans* biofilms on adhesive samples. **(a)** 24 h bacterial biofilm supernatant pH. **(b)** Bacterial biofilm lactic acid production. The means ± SD values. are shown, and different alphabetical markers represent significant intergroup differences (*P<* 0.05).

The pH of the supernatant of the modified adhesive samples adhering to the *S. mutans* biofilm after 24 h of incubation is shown in **Figure 9a**. The pH of the 0.5%, 1% and 2% groups (7.232 ± 0.033, 7.260 ± 0.014, and 7.263 ± 0.015, respectively) was not significantly different (*P* > 0.05) from that of the blank group (7.4 ± 0.01). The pH levels measured in the 0.05% and 0.1% groups were markedly lower than those in the blank and high-dose groups (*P*< 0.05), reaching 4.398 ± 0.128 and 4.814 ± 0.648, respectively. No statistically significant difference was observed relative to the 0% group (*P* > 0.05). The concentration of SCH-79797 at 0.5% significantly reduced the acid-producing capacity of the biofilm on the modified adhesive samples, with lactic acid yields (0.05%-2%) being 75.1%, 64.9%, 1.3%, 1.3%, 1.3%, and 0.1% of those in the 0% group (**Figure 9b**, *P*< 0.05).

## Discussion

In this study, we first verified the feasibility of SCH-79797-modified adhesive in terms of its bond strength, water contact angle, monomer double bond conversion rate, cytocompatibility and antimicrobial properties. We found that SCH-79797-modified adhesive (0.5%) maintained its adhesive properties and showed low cytotoxicity. It effectively inhibited *S. mutans* biofilm formation, reduced cariogenic virulence factors (EPS and lactic acid), and demonstrated potential to lower secondary caries risk at restoration margins, providing theoretical justification for its clinical application.

The incorporation of pharmacological agents can modify both the material characteristics of dental adhesives and the antibacterial performance of the incorporated agents, potentially influencing their clinical applicability. Currently, the testing of the bond strength of dental adhesives can be divided into shear force tests and tensile force tests. Depending on the use scenario, the choice of experimental method varies. Among many restorations, the main test object with tensile force is full crown restorations, whereas the main test object with shear force is resin restorations. The bond strength has been shown to vary as a function of the bonded surface area, particularly within the range of 0.25–11.65 mm^2^ (Sano et al., 1994). According to **Figure 2a**, both the commercial adhesive and the SCH-79797–containing adhesives exhibited shear strength values consistent with those obtained using De Munck’s method, without significant intergroup variation (*P* > 0.05). The shear strength values observed in this study fall within the empirically reported clinically acceptable range of approximately 20–30 MPa described in the literature (El Mourad, 2018). Notably, in the present work, only the bond strength following 24 h of water immersion was examined; therefore, additional aging assessments are needed to determine the practical durability of the adhesive system.

Monomer double bond conversion is an important parameter for the polymerization capacity of dental adhesives (Tjäderhane, 2015). The polymerization of methacrylate-based adhesives under light curing is governed by radical-mediated reactions that convert unsaturated C=C bonds into stable C–C bonds. The favorable double bond conversion rate increases the material’s tensile strength and hardness while decreasing its adsorption and solubility (Mokeem et al., 2022). A low double bond conversion rate results in a significant number of free monomers remaining in the bonding agent, reducing the bonding action. In this study, the double bond conversion rate of the modified adhesive significantly decreased when the concentration of SCH-79797 was greater than 1% (**Figure 2b**, *P*< 0.05). All the other SCH-79797 concentrations resulted in outcomes similar to those of the 0% control, and the differences did not reach statistical significance. (**Figure 2b**, *P* > 0.05). We speculate that the formation of linear polymer chains may have been disturbed when the SCH-79797 concentration was higher, which resulted in a lower double bond conversion rate (Nunes et al., 2005). The water contact angle is another critical physical property of the adhesive after curing. The hydrophilicity of the material increases as the water contact angle decreases, making it easier to cling to the biofilm (Sterzenbach et al., 2020). Similar to the double bond conversion results, the water contact angles of the 1% and 2% SCH-79797-modified adhesives were considerably less than those of the other groups (**Figure 2c**, *P*< 0.05). The reason could be that in the 1% and 2% groups, the comparatively low double bond conversion rate left more free monomers in the adhesive, and its hydrolyzable ester linkages could easily absorb water, resulting in increased hydrophilicity. In clinical applications, higher hydrophilicity and residual holes after the free monomers result in increased permeability, leading to faster adhesive degradation and increasing the risk of adhesive failure (Breschi et al., 2008).

The results of this study indicate that the release of SCH-79797 from Mold 2 exhibits distinct concentration-dependent and time-dependent characteristics. With the increase in initial drug loading concentration and the extension of immersion time, the cumulative release amount increased significantly. This phenomenon is consistent with the passive diffusion mechanism commonly observed in polymer-based drug release systems, in which the incorporated drug diffuses from the high-concentration interior of the matrix into the low-concentration external release medium. Accordingly, a greater concentration gradient provides a stronger driving force for diffusion resulting in increased drug release.

On the basis of the results of the CCK-8 assays, the viability of L929 cells after 24 h of culture initially increased and subsequently decreased with increasing drug fraction (**Figure 4a**). Moreover, higher SCH-79797 concentrations may result in increased drug release into the culture medium, thereby contributing to enhanced cytotoxicity. Nevertheless, the survival rate of L929 cells in all adhesive sample groups remained above 75%, and in some groups even exceeded 100%, meeting clinical acceptance criteria (Gong et al., 2019). The live/dead cell staining images revealed that when the SCH-79797 concentration was below 0.5%, L929 cells grew well, with only a few scattered dead cells observed. In contrast, when the concentration reached 1% and above, the number of viable cells decreased while dead cells gradually increased. These findings suggest that the cytotoxicity threshold of the SCH-79797-modified adhesive toward L929 cells is approximately 0.5%, below which no apparent cytotoxic effects were observed. Notably, even at a high concentration of 2%, the death rate remained at a relatively low level, suggesting that this extract has favorable biocompatibility, highlighting the potential of the modified adhesive in clinical applications.

Consistent with our previous findings, the cured SCH-79797–modified adhesive in the present study effectively inhibited *S. mutans* biofilm formation (Zhang et al., 2022b). As presented in **Figure 5a**, compared with the control, increasing concentrations of SCH-79797 within the adhesive progressively reduced biofilm formation. SCH-79797 retains its antibacterial activity after adhesive curing because these compounds are soluble in hydrophobic dental monomers, are uniformly dispersed in the resin matrix, and are minimally involved in polymerization reactions (Cheng et al., 2015). As a result, a modest concentration of the chemical has a powerful antibacterial effect while preserving the mechanical qualities of the adhesive. Similarly, the results of MTT experiments revealed that the modified adhesive has substantial antibiofilm activity, as it is a yellow compound that can be converted to a water-insoluble blue–violet molecule by succinate dehydrogenase in living cell mitochondria but not in dead cells (Benov, 2021). SCH-79797–modified adhesives at concentrations of 0.5% and 1% effectively suppressed *S. mutans* biofilm viability, with effects comparable to those observed at higher concentrations. The lack of detectable metabolic activity suggests a pronounced impairment of biofilm function after the incorporation of SCH-79797 into the adhesive. The minor differences observed between the 0.5% and 1% groups may be related to reduced assay sensitivity at low bacterial levels or potential interference of SCH-79797 with bacterial metabolic enzymes. Previous studies have described SCH-79797 as a pyrroloquinoline azodiamine–based molecule featuring an isopropyl substituent at one terminus and a phenyl moiety bearing a cyclopropyl group at the opposite end, in which the pyrroloquinoline azodiamine core targets the activity of dihydrofolate reductase; thus, whether this drug affects succinate dehydrogenase in *S. mutans* deserves further investigation (Martin et al., 2020). SEM analysis of the bacterial biofilm revealed that bacterial extracellular polysaccharide secretion was greatly decreased and that the cellular morphology was wrinkled or even lysed in the groups modified with high concentrations of SCH-79797 (0.5%, 1%, and 2%). These findings further support another antibacterial mechanism of SCH-79797, which involves targeting the isopropylbenzene moiety to affect cell membrane integrity (Martin et al., 2020). Live/dead bacterial staining revealed that the bacteria in the 0.5% group were predominantly nonviable. Consistent with these findings, biofilm CFU analysis demonstrated that incubation with the 0.5% SCH-79797–modified adhesive led to a pronounced decrease in viable bacterial counts within the biofilm after 24 h. (**Figure 5a**). In the initial experiments, biofilm formation was still observed at lower SCH-79797 concentrations in the culture medium, suggesting that the 0.5% modified adhesive may have released a higher effective drug concentration within the culture system during the 24-h incubation period. Nevertheless, further drug release studies are needed to verify this assumption. Although bacterial biofilms could still be detected on the surfaces of the 1% and 2% modified adhesives by SEM, CLSM revealed no visible live/dead bacterial staining signals (**Figure 6**, **7**). This discrepancy is likely due to the markedly diminished bacterial presence in these groups, together with the intrinsic autofluorescence of the adhesive resin, which likely interfered with fluorescence-based bacterial observation.

Composite resin restorations are widely used; however, degradation of the adhesive interface creates a favorable microenvironment for cariogenic biofilms (Geraldeli et al., 2017). Bacterial acid production and EPS synthesis play central roles in this process by promoting demineralization, biofilm stabilization, and further degradation of the adhesive interface (Mennito et al., 2022). Therefore, strategies that reduce lactic acid and EPS production at the adhesive surface are indispensable for prolonging the durability and clinical longevity of resin-bonded restorations. Extracellular polysaccharides (EPS) constitute the polysaccharide component of the biofilm matrix, comprising both water-soluble and water-insoluble fractions (Rudin et al., 2023). Among these, water-insoluble glucans (WIG), a major component of the insoluble extracellular polysaccharide matrix, are a key structural component that contributes to the mechanical stability of biofilms. In the present study, a glucan-specific fluorescent dye was employed to detect glucans within the biofilm matrix. It should be noted that WIG encompasses a broader category of water-insoluble polysaccharides, whereas the staining approach used here specifically targets glucan components rather than all WIG constituents. In this study, compared with the control, SCH-79797–modified adhesives markedly suppressed the production of EPS and WIG, indicating a substantial attenuation of biofilm structural integrity. The reduced EPS production may be partly attributable to the pronounced decrease in bacterial biomass and to stress-induced metabolic adaptation, whereby bacteria prioritize survival over polysaccharide synthesis (Zhang et al., 2015a). In addition, the reduction in EPS production observed in this study may be associated with alterations in the enzymatic processes involved in EPS synthesis. In *S. mutans*, EPS production is primarily mediated by glucosyltransferases, which play a key role in polysaccharide formation. Therefore, the decreased EPS levels may be related to potential effects of SCH-79797 on glucosyltransferase activity. However, this hypothesis remains to be further experimentally validated. In parallel, SCH-79797 significantly mitigated the acidogenicity of *S. mutans* biofilms, as reflected by elevated biofilm supernatant pH values above the demineralization threshold and a marked reduction in lactic acid production (Touger-Decker and van Loveren, 2003). The decrease in lactic acid production observed in the SCH-79797-modified adhesive groups may be attributed to multiple factors. The reduced bacterial biomass induced by SCH-79797 could directly lead to lower overall acid production. In addition, lactic acid generation in *S. mutans* is primarily mediated by lactate dehydrogenase, and the observed reduction may also reflect a potential influence of SCH-79797 on LDH-related metabolic processes. However, the relative contribution of these factors remains to be further elucidated. Collectively, these effects suggest that SCH-79797 not only limits biofilm accumulation but also effectively attenuates key cariogenic virulence factors, thereby potentially improving the integrity of the adhesive interface.

Nevertheless, this study has several limitations that warrant consideration. First, although the SCH-79797-modified adhesive exhibited pronounced antibacterial activity in the short term, its long-term antimicrobial efficacy remains to be clarified, particularly given that drug release kinetics represent a critical concern. Second, this study used a single-species *S. mutans* biofilm model, which does not fully recapitulate the complex polymicrobial environment of the oral cavity, where multiple bacterial species interact. Third, the potential impact of SCH-79797-modified adhesives on oral commensal bacteria, such as *Streptococcus sanguinis*, has not been systematically evaluated in this context. Future work will aim to optimize the chemical modification of the adhesive to achieve sustained antimicrobial effects while preserving bonding performance, and to evaluate its effects in multispecies biofilm models and on oral commensal flora. In addition, animal studies evaluating caries prevention and biosafety will be necessary to further validate the clinical potential of this strategy.

## Conclusion

0.5% or less SCH-79797-modified adhesive did not significantly change the adhesive properties, and the modified adhesive had good biocompatibility. SCH-79797-modified adhesive can effectively suppress *S. mutans* biofilm development and reduce the expression of its cariogenic virulence factors, EPS and lactic acid, indicating a potential protective effect against secondary caries at the edges of composite resin restorations. These experimental results provide additional theoretical justification for the clinical use of SCH-79797-modified adhesives.

## Data Availability

The original contributions presented in the study are included in the article/supplementary material. Further inquiries can be directed to the corresponding authors.
